# Systematical Analysis of the Cancer Genome Atlas Database Reveals *EMCN*/*MUC15* Combination as a Prognostic Signature for Gastric Cancer

**DOI:** 10.3389/fmolb.2020.00019

**Published:** 2020-02-25

**Authors:** Wentao Dai, Jixiang Liu, Bingya Liu, Quanxue Li, Qingqing Sang, Yuan-Yuan Li

**Affiliations:** ^1^Shanghai Center for Bioinformation Technology, Shanghai, China; ^2^Shanghai Key Laboratory of Gastric Neoplasms, Department of Surgery, Shanghai Institute of Digestive Surgery, Ruijin Hospital, Shanghai Jiao Tong University School of Medicine, Shanghai, China; ^3^Shanghai Engineering Research Center of Pharmaceutical Translation, Shanghai Industrial Technology Institute, Shanghai, China; ^4^School of Biotechnology, East China University of Science and Technology, Shanghai, China

**Keywords:** MUC family, EMCN, MUC15, prognostic, gastric cancer

## Abstract

Digestive cancers-including gastric cancer (GC), colorectal cancer, hepatocellular carcinoma, esophageal cancer, and pancreatic cancer-accounted for 26% of cancer cases and 35% of cancer deaths worldwide in 2018. It is crucial and urgent to develop biomarkers for the diagnosis, prognosis, and therapeutic benefits of digestive cancers, especially for GC, since the incidence of GC is lower only than lung cancer in China, is hard to detect at an early stage, and is associated with poor prognosis. Mucins, glycoproteins encoded by MUC family genes, act as a part of a physical barrier in the digestive tract and participate in various signaling pathways. Some mucins have been used or proposed as biomarkers for carcinomas, such as MUC16 (CA125) and MUC4. However, there are no systematic investigations on the association of MUC family members with diagnoses and clinical outcomes even though relevant data have been largely accumulated in the past decade. By analyzing transcriptomic and clinical data of digestive cancer samples from TCGA involving colon adenocarcinoma (COAD), esophageal carcinoma (ESCA), liver hepatocellular carcinoma (LIHC), stomach adenocarcinoma (STAD), and pancreatic adenocarcinoma (PAAD), it was found that expressions levels of *MUC15*, *MUC13*, and *MUC21* were individually associated with survival for digestive cancers, and high expressions of *EMCN* (MUC14) and *MUC15* were correlated with poor survival for STAD. Cox regression analysis indicated the predictive power of an *EMCN*/*MUC15* combination for overall survival (OS) of GC patients, which was validated on an independent dataset from GEO. EMCN/MUC15 correlated genes were identified to be enriched in cancer-related processes, such as vasculature development, mitosis, and immunity. Therefore, we propose that an *EMCN*/*MUC15* combination could be a potential prognostic signature for gastric cancer.

## Introduction

Digestive cancers are a group of cancers that occur in the digestive tract, and include gastric cancer (GC), colorectal cancer, hepatocellular carcinoma, esophageal cancer, and pancreatic cancer. Digestive cancers accounted for around 26% of cancer cases and 35% of cancer deaths in the world in 2018 (Bray et al., 2018). Among them, the morbidity and mortality of GC in Eastern Asia is much higher than the worldwide average level. In China, the incidence of GC is only lower than lung cancer, and the mortality is third to lung cancer and liver cancer (Chen et al., 2014). Most patients suffering from early stage GC are asymptomatic and always develop distant metastasis at the time of diagnosis (Van Cutsem et al., 2016; Bray et al., 2018). Surgery is the main treatment for GC. Adjuvant or neoadjuvant therapy combined with surgery is commonly used to treat advanced GC, while targeted drugs for advanced GC, such as the HER2 (also known as ERBB2) antibody trastuzumab, and the VEGFR-2 antibody ramucirumab, are still in clinical trials (Van Cutsem et al., 2016). Therefore, developing biomarkers for the diagnosis, prognosis, and therapeutic response of digestive cancers, especially of GC, is necessary and urgent for reducing the mortality rate.

Mucins represent a group of glycoproteins encoded by MUC family genes. These high-molecular weight and filamentous glycoproteins could be classified into secreted mucins and membrane-bound mucins. In the digestive tract, secreted mucins form a mucus layer and act as part of a physical defensive barrier against external aggressive forces (Dekker et al., 2002; Dhanisha et al., 2018); membrane-bound mucins possess membrane specific domains which enable their diverse roles in signaling pathways (Dekker et al., 2002; Dhanisha et al., 2018). Not surprisingly, dysfunction of mucins in their fundamental roles is implicated in disease development at mucosal surfaces (Corfield, 2015; Dhanisha et al., 2018), and some mucins have been reported to display diagnostic or prognostic significance in different types of cancer. For example, MUC16, also known as CA125, is a widely used biomarker for the diagnosis of ovarian cancer (Yonezawa et al., 2011; Jonckheere and Van Seuningen, 2018) and was also found to be over-expressed in several other human malignancies, including pancreas, breast, and lung (Aithal et al., 2018). MUC4 promotes carcinogenetic progression and has been proposed as a promising biomarker for pancreatic, ovarian, esophagus, and lung cancers (Kaur et al., 2013; Jonckheere and Van Seuningen, 2018). MUC15 overexpression is significantly correlated with several types of cancers, including colon cancer, hepatocellular carcinoma, and thyroid cancer (Huang et al., 2009; Nam et al., 2011; Wang et al., 2013; Choi et al., 2018). Moreover, *MUC4*/*MUC16*/*MUC20* high-expression signature was very recently reported to be correlated with poor overall survival (OS) in several types of digestive cancers including pancreatic, colon, and GCs (Jonckheere and Van Seuningen, 2018). However, there are no systematic investigations, so far, on the association of MUC family members with diagnosis, prognosis, and/or therapeutic benefits, even though the Cancer Genome Atlas (TCGA) project is producing massive genomic, transcriptomic, proteomic, and clinical data involving more than 11,000 patients of 33 different types of tumors (Weinstein et al., 2013), and meanwhile, a number of web tools, such as GEPIA (Tang et al., 2017) and cBioPortal for Cancer Genomics (Cerami et al., 2012; Gao et al., 2013), have been developed that enable users to easily and effectively mine TCGA data.

In the present study, by analyzing digestive cancer samples from TCGA involving colon adenocarcinoma (COAD), esophageal carcinoma (ESCA), liver hepatocellular carcinoma (LIHC), stomach adenocarcinoma (STAD), and pancreatic adenocarcinoma (PAAD), we found that expression levels of *MUC15*, *MUC13*, and *MUC21* were individually associated with survival for all these digestive cancers, and high expressions of *EMCN* (MUC14) and *MUC15* were correlated with poor survival for STAD. Cox regression analysis showed that *EMCN*/*MUC15* combination still exhibited a significant correlation with the OS of GC patients. The prognostic prediction power of signature *EMCN*/*MUC15* was further validated on an independent GC dataset, GSE84437. *EMCN*/*MUC15* top 50 correlated genes were identified to be enriched in cancer-related processes, including vasculature development, mitosis, immunity, and so on. Taken together, we propose *EMCN*/*MUC15* combination as a potential prognostic signature for GC.

## Materials and Methods

### Datasets

Datasets were collected from TCGA^1^ and GEO^2^ (Barrett et al., 2012). Specifically, gene expression data (TPM, Transcripts Per Kilobase Million) and clinical data for digestive cancers including COAD, ESCA, LIHC, STAD, and PAAD, were analyzed with the online webserver GEPIA 1.0 (Tang et al., 2017). Among them, MUC family mRNA expression data (mRNA expression z-scores, which is based on RNASeqV2 processed and normalized using RSEM) and clinical profiles involving 407 STAD samples were extracted by using an online web tool cBioPortal for Cancer Genomics (Cerami et al., 2012; Gao et al., 2013). Additionally, GSE84437 were extracted from the GEO database, which involves mRNA microarray data and clinical profiles of 433 GC samples.

### Survival Analysis

Kaplan–Meier (KM) survival analysis for digestive cancer samples as a whole was carried out by using the webserver GEPIA 1.0 (Tang et al., 2017), and for GC samples (TCGA-STAD from cBioPortal and GSE84437 R package *survival*^3^ was used. KM analysis was based on individual gene expression value and survival data. By using the median expression value of a query gene in a certain sample group as a cutoff, the samples were split into high and low expression groups with the expression level of the query gene not less than and less than the cutoff. The Cox proportional hazard model was built by using R package *survival*, fitted with two genes’ expression values for OS or disease free survival (DFS). Similar to the individual gene analysis, the median value of weighted expression value (shortened as WEV) of a gene combination in a certain cohort were used as a group cutoff, where WEV was calculated as the sum of cox-regression coefficient weighted expression value of each gene involved in the combination. Log rank *p*-values, cox proportional hazard ratios (HRs), and HR *p*-values were calculated to compare the survival between two groups split by the median value of gene expression or WEV. A *p*-value of less than 0.05 and HR greater than 1.05 or less than 0.95 suggest statistical significance of the survival difference between high and low groups, which indicates the corresponding gene or gene combination has a prognostic potential.

### Gene Co-expression Analysis and Enrichment Analysis

Gene co-expression analysis was carried out using webserver cBioPortal, and the top 25 positively correlated and top 25 negatively correlated genes were selected according to Spearman correlation coefficients, which were taken together and simplified as “top 50 correlated genes” in our results. Here, correlated genes met two criteria: the absolute value of Spearman correlation coefficient is greater than 0.25, and the *p*-value is less than 0.01. Gene set enrichment analysis (GSEA) was performed by using R package *clusterProfiler* (Yu et al., 2012). The pathways enriched for GO (Gene Ontology) (Ashburner et al., 2000; The Gene Ontology Consortium, 2019) were plotted based on the negative logarithm of *p*-value.

## Results

### MUC15, 13, and 21 Display Prognostic Potential for Digestive Cancer on TCGA

Aiming to assess the prognostic potentials of every MUC gene, KM survival analysis was applied to TCGA digestive cancer samples as a whole involving COAD, ESCA, LIHC, STAD, and PAAD by using the webserver GEPIA 1.0 (Tang et al., 2017). Among the 14 MUC family members with expression data available, the expression levels of MUC1, MUC5AC, MUC6, OVGP1 (MUC9), MUC13, EMCN (MUC14), MUC15, MUC16, MUC17, and MUC21 individually exhibited significant correlations with OS, with HR *p*-values less than 0.05 and HR greater than 1.05 or less than 0.95; similarly, MUC2, MUC3A, MUC12, MUC13, MUC15, MUC17, MUC20, and MUC21 were significantly correlated with DFS (Table 1 and Supplementary Figure S1). *MUC13*, *MUC15*, *MUC17*, and *MUC21* were significant for both OS and DFS, among which MUC15 performed best for OS correlation and the second best for DFS correlation. In comparison, MUC13 displayed the best performance in DFS analysis, while ranked relatively lower (9th) in OS analysis; MUC21 ranked 3rd for OS, and 8th for DFS (Table 1 and Supplementary Figure S1). These indicate that *MUC15* represents a promising candidate for developing strategies for prognosis prediction for digestive cancers.

**TABLE 1:** Survival analysis of TCGA digestive cancer samples for prognostic potentials of MUC family genes.

**Gene**	**HR *p*-value for OS**	**OS *p*-value rank**	**HR *p*-value for DFS**	**DFS *p*-value rank**
MUC1	**1.3E−05**	6	0.23	11
MUC2	0.69	14	**5.2E−08**	3
MUC3A	0.49	12	**1.7E−06**	4
MUC5AC	**7.9E−06**	5	0.74	14
MUC6	**2.7E−07**	3	0.41	12
OVGP1 (MUC9)	**0.0021**	7	0.094	10
MUC12	0.58	13	**0.00012**	5
MUC13	**0.032**	9	**2.1E−08**	1
EMCN (MUC14)	**0.044**	10	0.71	13
MUC15	**1.7E−09**	1	**3.6E−08**	2
MUC16	**6.4E−09**	2	0.059	9
MUC17	**0.0053**	8	**0.00051**	6
MUC20	0.36	11	**0.01**	7
MUC21	**9.8E−07**	4	**0.01**	8

*OS stands for overall survival and DFS stands for disease free survival (DFS). The *p*-values less than 0.05 are displayed in bold.*

### MUC14 (EMCN) and 15 Display Prognostic Potential for Gastric Cancer on TCGA-STAD

**FIGURE 1:**
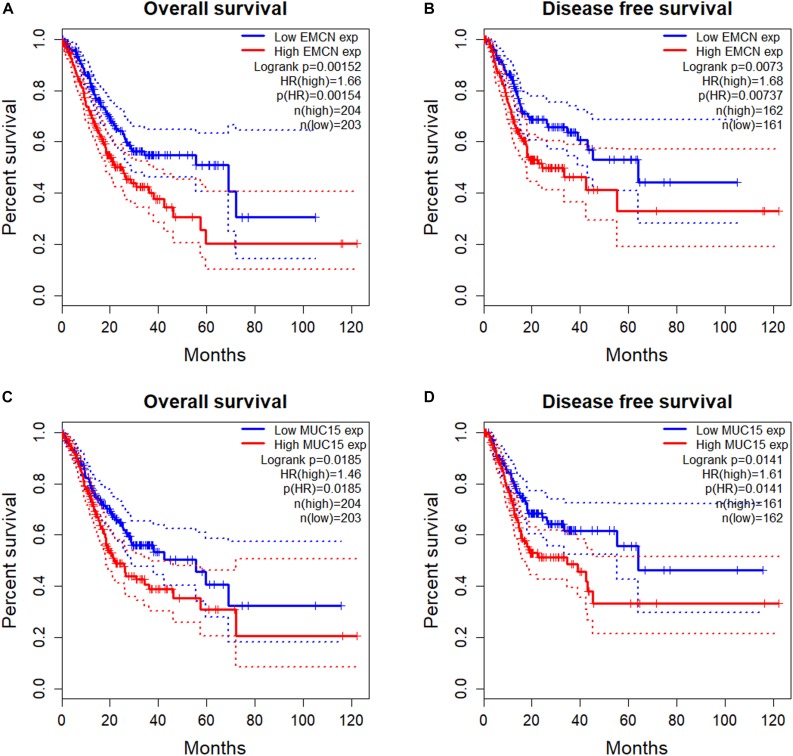
Survival analysis of TCGA STAD samples for prognostic potentials of EMCN (MUC14) and MUC15. **(A)** Overall Survival (OS) of EMCN. **(B)** Disease Free Survival (DFS) of EMCN. **(C)** Overall Survival of MUC15. **(D)** Disease Free Survival of MUC15. Log rank *p*-values, hazard ratios (HRs) and hazard ratio *p*-values were calculated. The 95% confidence intervals for survival time were shown in as dotted lines in the Kaplan–Meier (KM) survival plot.

To investigate the prognostic potentials of MUC family genes for STAD, we performed KM survival analysis exclusively on STAD samples from TCGA with R package *survival*. It was found that the expression levels of *EMCN* (MUC14) and *MUC15* individually showed significant correlations with both OS and DFS, and MCAM (MUC18) was significant only with OS (Table 2). KM survival plots, together with log rank *p*-values, cox proportional HRs, and HR *p*-values summarized in Figure 1 indicated that *EMCN* performed better than *MUC15* in both OS and DFS analyses. Overall, *EMCN* and *MUC15* could be potential biomarkers for STAD prognosis.

**TABLE 2:** Survival analysis of TCGA STAD samples for prognostic potentials of MUC family genes.

**Gene**	**HR *p*-value for OS**	**HR *p*-value for DFS**
MUC1	0.654	0.591
MUC2	0.129	0.364
MUC4	0.9	0.203
MUC5B	0.441	0.753
MUC6	0.67	0.0854
OVGP1 (MUC9)	0.662	0.925
MUC12	0.957	0.637
MUC13	0.0511	0.234
EMCN (MUC14)	**0.00154**	**0.00737**
MUC15	**0.0185**	**0.0141**
MUC16	0.825	0.0975
MUC17	0.145	0.406
MCAM (MUC18)	**0.0167**	0.323
MUC20	0.891	0.62
MUC21	0.224	0.745

*OS stands for overall survival and DFS stands for disease free survival. The *p*-values less than 0.05 are displayed in bold.*

### *EMCN/MUC15* Combination Could Serve as Prognostic Signature for Gastric Cancer

**FIGURE 2:**
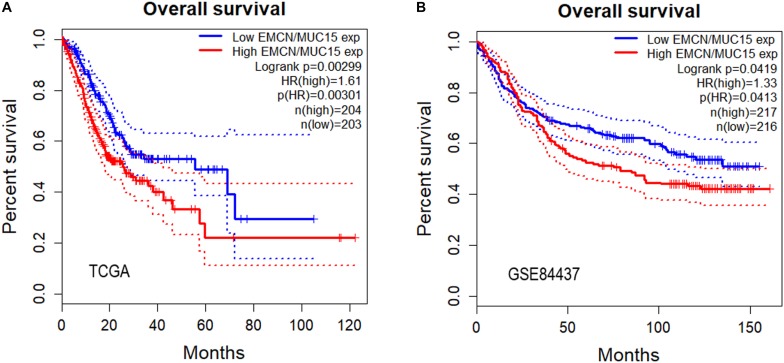
Overall survival analyses of gastric cancer (GC) samples from TCGA STAD **(A)** and GSE84437 **(B)** for predictive power of EMCN/MUC15 signature. Log rank *p*-values, hazard ratios (HRs) and hazard ratio *p*-values were calculated. The 95% confidence intervals for survival time were shown as dotted lines in the Kaplan–Meier survival plot.

So far we have observed that high expressions of both EMCN and MUC15 were associated with poor prognosis in GC, and that EMCN and MUC15 displayed the strongest correlation to survival for GC and digestive cancers, respectively (Table 2 and Figure 1). Thus, we set out to investigate whether *EMCN*/*MUC15* combination could be a prognostic signature for GC. Cox proportional hazards regression analysis was performed based on the two genes’ expression values and OS data derived from TCGA STAD dataset. As expected, the expression of *EMCN*/*MUC15* combination exhibited significant correlation with OS, with log rank *p*-value of 0.00299 and HR *p*-value of 0.00301 (Figure 2A).

We then separately tested the prognostic prediction power of *EMCN*, *MUC15* and their combination on an independent dataset, GSE84437, which involved 433 GC samples. Again, significant results of *EMCN*/*MUC15* combination (HR = 1.33) were obtained with log rank *p*-value being 0.0419 and HR *p*-value being 0.0413 (Figure 2B); while one single gene, *EMCN* (HR *p*-value of 0.0807, HR = 1.27) or *MUC15* (HR *p*-value of 0.156, HR = 0.82), had no significant prognostic prediction power, as shown in Supplementary Figure S2. We therefore proposed that *EMCN*/*MUC15* combination could be a potential prognostic signature for GC.

### *EMCN/MUC15* Correlated Genes Are Functionally Enriched in Cancer Related Processes

By using webserver cBioPortal, the top 50 EMCN- (Table 3) or MUC15- (Table 4) correlated genes were identified based on mRNA expression data of TCGA STAD samples, including the top 25 positively correlated genes and top 25 negatively correlated genes. It is noticeable that there is no intersection between the two top 50 gene lists at all and no co-expression between *EMCN* and *MUC15* (Spearman’s Correlation of 0.0264 with *p*-value of 0.592) either, implying the functional complementarity between *EMCN* and *MUC15* and thus the rationality of the combination of the two genes in predicting prognosis for GC.

**TABLE 3:** Top 50 genes correlated with EMCN based on TCGA STAD dataset.

**Correlated gene**	**Cytoband**	**Spearman correlation**	***p*-value**
CYYR1	21q21.3	0.931414	2.19E−183
MYCT1	6q25.2	0.929044	1.90E−180
ERG	21q22.2	0.894179	3.19E−146
DIPK2B	Xp11.3	0.887525	4.57E−141
ADGRL4	1p31.1	0.886757	1.71E−140
***CD34***	1q32.2	0.880383	6.99E−136
***TEK***	9p21.2	0.873397	4.03E−131
***PECAM1***	17q23.3	0.871639	5.73E−130
S1PR1	1p21.2	0.870224	4.72E−129
LDB2	4p15.32	0.860092	8.59E−123
RHOJ	14q23.2	0.859913	1.10E−122
CLEC14A	14q21.1	0.854201	2.25E−119
GNG11	7q21.3	0.853027	1.03E−118
EBF1	5q33.3	0.846286	5.16E−115
MMRN2	10q23.2	0.846005	7.29E−115
CLEC1A	12p13.2	0.843416	1.71E−113
CALCRL	2q32.1	0.841594	1.53E−112
LRRC70	5q12.1	0.84015	8.47E−112
MEF2C	5q14.3	0.839354	2.16E−111
ARHGEF15	17p13.1	0.836065	9.86E−110
CDH5	16q21	0.828483	4.80E−106
PALMD	1p21.2	0.828283	5.97E−106
SHE	1q21.3	0.826792	3.01E−105
SPARCL1	4q22.1	0.823121	1.52E−103
JAM2	21q21.3	0.821442	8.85E−103
RAD54L	1p34.1	–0.53926	1.10E−32
CDCA5	11q13.1	–0.53612	2.96E−32
PKP3	11p15.5	–0.53108	1.41E−31
CDCA8	1p34.3	–0.5303	1.79E−31
ZWINT	10q21.1	–0.52817	3.44E−31
KIF2C	1p34.1	–0.52339	1.46E−30
HJURP	2q37.1	–0.51982	4.21E−30
MCM2	3q21.3	–0.51829	6.63E−30
CDT1	16q24.3	–0.51369	2.54E−29
MYO19	17q12	–0.51058	6.24E−29
TONSL	8q24.3	–0.50684	1.82E−28
CCNA2	4q27	–0.5056	2.58E−28
NCAPH	2q11.2	–0.5018	7.48E−28
POC1A	3p21.2	–0.50165	7.81E−28
NELFA	4p16.3	–0.50116	8.95E−28
UBE2T	1q32.1	–0.50026	1.15E−27
POLD2	7p13	–0.49997	1.25E−27
DTL	1q32.3	–0.49967	1.35E−27
PTBP1	19p13.3	–0.49959	1.38E−27
CNOT11	2q11.2	–0.49871	1.76E−27
STIP1	11q13.1	–0.49718	2.69E−27
MAP7	6q23.3	–0.49631	3.41E−27
ESPL1	12q13.13	–0.49591	3.81E−27
TBRG4	7p13	–0.49548	4.29E−27
CDC25A	3p21.31	–0.49474	5.24E−27

*Genes mentioned in Discussion section are highlighted in bold and italic.*

**TABLE 4:** Top 50 genes correlated with MUC15 based on TCGA STAD dataset.

**Correlated gene**	**Cytoband**	**Spearman correlation**	***p*-value**
ANO3	11p14.3-p14.2	0.558879	1.82E−35
FSTL4	5q31.1	0.4959	3.82E−27
TMPRSS13	11q23.3	0.469609	3.76E−24
ZNF750	17q25.3	0.464898	1.21E−23
LGALS7	19q13.2	0.454428	1.54E−22
NCCRP1	19q13.2	0.452369	2.52E−22
PCLO	7q21.11	0.449054	5.50E−22
GABRA3	Xq28	0.446711	9.51E−22
DLX3	17q21.33	0.443637	1.94E−21
LIN28B	6q16.3-q21	0.440243	4.21E−21
ADGRV1	5q14.3	0.439028	5.55E−21
USH1G	17q25.1	0.436641	9.52E−21
C12ORF56	12q14.2	0.429849	4.32E−20
RSPO4	20p13	0.428819	5.41E−20
SPAG17	1p12	0.425992	1.00E−19
MARK1	1q41	0.424353	1.43E−19
HTR2C	Xq23	0.423044	1.90E−19
CT45A5	Xq26.3	0.420712	3.13E−19
PRPF40B	12q13.12	0.419994	3.64E−19
C3ORF67	3p14.2	0.419376	4.16E−19
RIPPLY3	21q22.13	0.417437	6.27E−19
CNGB3	8q21.3	0.417398	6.32E−19
ATP6V0A4	7q34	0.413452	1.45E−18
LINC00964	8q24.13	0.412548	1.74E−18
VGLL1	Xq26.3	0.409463	3.30E−18
MCUB	4q25	–0.35985	3.92E−14
FAS	10q23.31	–0.32779	7.51E−12
IRF1	5q31.1	–0.32732	8.08E−12
ZIC2	13q32.3	–0.31402	5.99E−11
CDC42SE2	5q31.1	–0.31243	7.55E−11
HK3	5q35.2	–0.30198	3.37E−10
NUB1	7q36.1	–0.30007	4.41E−10
GBP4	1p22.2	–0.29733	6.45E−10
BBC3	19q13.32	–0.29722	6.55E−10
AIM2	1q23.1-q23.2	–0.29707	6.68E−10
NLRC5	16q13	–0.29669	7.04E−10
MAX	14q23.3	–0.29642	7.30E−10
MTHFD1	14q23.3	–0.29437	9.67E−10
AGAP2	12q14.1	–0.29096	1.54E−09
***IFNG***	12q15	–0.29068	1.59E−09
RASSF1	3p21.31	–0.28787	2.32E−09
GZMA	5q11.2	–0.28696	2.62E−09
CCL4	17q12	–0.28515	3.32E−09
MAT2B	5q34	–0.28231	4.82E−09
FCGR3A	1q23.3	–0.28226	4.85E−09
THG1L	5q33.3	–0.28207	4.97E−09
TK2	16q21	–0.28202	5.01E−09
PRKX	Xp22.33	–0.27772	8.71E−09
JAK2	9p24.1	–0.27752	8.94E−09
EEF2	19p13.3	–0.2774	9.07E−09

*Genes mentioned in Discussion section are highlighted in bold and italic.*

**FIGURE 3:**
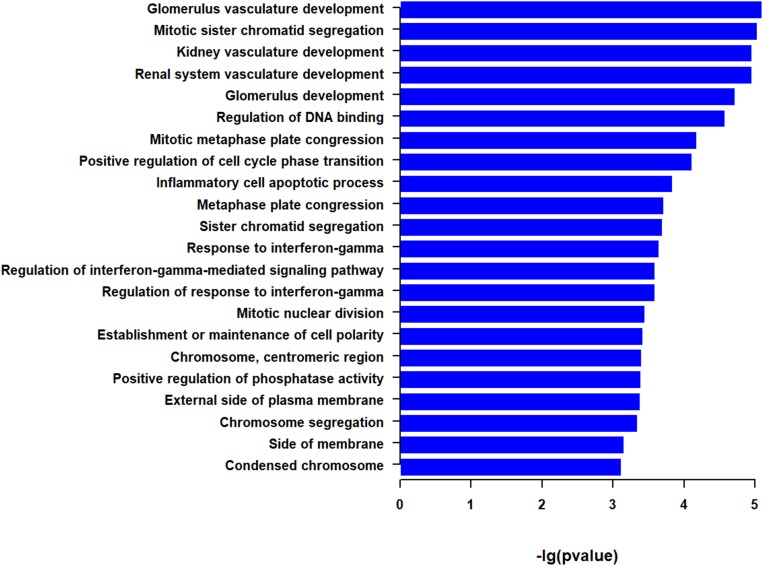
Go terms derived from gene set enrichment analysis (GSEA) for top 50 EMCN- and MUC15-correlated genes. The pathways are ranked by -log *p*-value. The 95% confidence intervals for survival time were shown as dotted lines in the Kaplan–Meier survival plot.

We then performed functional enrichment analysis with the two top 50 correlated genes as a whole. GSEA identified a total of 22 GO terms (Figure 3 and Supplementary Table S1). Among them, the most significant pathways were associated with vasculature development, such as glomerulus vasculature development and renal system vasculature development. Some enriched pathways are associated with mitosis, such as mitotic sister chromatid segregation and mitotic metaphase plate congression. Some pathways were associated with immunity, such as inflammatory cell apoptotic process and response to interferon-gamma. The other enriched pathways were involved in DNA binding, cell cycle phase transition, cell polarity, phosphatase activity, and side of plasma membrane (Figure 3 and Supplementary Table S1). These indicate that genes correlated with EMCN and MUC15 in GC tend to be enriched in cancer related processes, such as vasculature development, mitosis, and immunity.

## Discussion

In the present study, by systematically analyzing mRNA expression and clinical data of TCGA digestive cancer samples and GEO GC samples, we propose MUC15 as a promising candidate for prognosis prediction of digestive cancers, and *EMCN*/*MUC15* combination as a potential prognostic signature for GC.

Gene signature identification is essentially a process of dimension reduction of high dimensional data. On one hand, a signature involving less features or genes obviously has more practicality; on the other hand, a signature is also expected to have sufficient interpretability, although it is far from achieved. In this sense, a good signature is supposed to consist of orthogonal or mutually exclusive features which are able to hold a testable hypothesis from a systematic viewpoint while also sustaining the robustness and reliability of the signature. However, most current efforts in this field focus on reducing dimension over enhancing explanatory power of the signature. In our work, although *EMCN* and *MUC15* coding genes belong to the same gene family, it is noted that there is no expression correlation between the two genes and no intersection between their top 50 correlated genes, implying the orthogonality and functional complementarity between EMCN and MUC15. As we expected, the combination of *EMCN*/*MUC15* shows more robust prognostic power than the individual genes in GC according to the testing result implemented on an independent dataset GSE84437. These observations not only support the rationality of the combination of the two genes in predicting prognosis, but also indicate the explanatory power of *EMCN*/*MUC15* signature, which is supposed to play an important role in the robustness improvement.

*EMCN*, i.e. MUC14, encodes a membrane-bound protein, endothelial sialomucin or mucin-like sialo glycoprotein, which was reported to inhibit cell and extracellular matrix interaction, interfere with leukocyte-endothelial cell adhesion, and even promote the peritoneal metastasis process of GC cells (Liu et al., 2001; Zahr et al., 2016; Dhanisha et al., 2018; Bao et al., 2019). Among the 22 enriched functions for top 50 EMCN-correlated genes and top 50 MUC15-correlated genes, the most significant one is glomerulus vasculature development that is associated with four *EMCN*/*MUC15* correlated genes including *CD34*, *TEK*, *PECAM1*, and *IFNG* (Tables 3, 4 and Supplementary Table S1). After carefully checking functional annotations of the four genes, we focused on two cancer relevant genes, *CD34* and *PECAM1*. Both genes are significantly coexpressed with *EMCN* with correlation coefficients of 0.880 and 0.871, respectively (Table 3). *CD34*, a marker of vascular endothelial cells, is capable of supporting cell adhesion by increasing surface expression (Nielsen and McNagny, 2008). *PECAM1*, also known as *CD31*, encodes platelet endothelial cell adhesion molecule 1 that is necessary for leukocyte transendothelial migration (TEM) (Dasgupta et al., 2009). It is noteworthy that *EMCN*/*COL4A5*/*CCL11* combination was very recently reported as a prognostic signature for diffuse type GC (Bao et al., 2019). In our study, among MUC family members, *EMCN* exhibits the strongest correlation with survival for GC. Taken together, *EMCN* may play crucial roles in tumorigenesis and progression of GC via cell adhesion and TEM of lymphocytes.

*MUC15* also encodes a membrane-bound protein, which could promote cell proliferation, cell-extracellular matrix adhesion, colony forming ability, and invasion in colon cancer cells (Huang et al., 2009). Its overexpression is significantly correlated with diverse cancers (Pallesen et al., 2002; Shyu et al., 2007; Huang et al., 2009; Nam et al., 2011; Wang et al., 2013; Choi et al., 2018). However, it was also found that the expression of *MUC15* decreased in hepatocellular carcinoma cells and negatively regulated metastasis of hepatocellular carcinoma (Wang et al., 2013). This suggests that *MUC15* may perform diverse functions in tumorigenesis and progression. In our study, *MUC15* displays the strongest correlation among the MUC family with survival for digestive cancers and *MUC15* overexpression seems to be a promising candidate for a prognosis biomarker of digestive cancers. Combined with *EMCN*, the two genes provide a potential prognostic signature for GC and show more robustness in the prognostic prediction power than individual genes. As far as we know, the association of *MUC15* with GC is rarely reported.

In summary, we propose *EMCN*/*MUC15* combination as a prognostic signature with mechanistic interpretability. It not only possesses prognostic capability for GC, but also offers clues for further exploring systematic mechanisms of carcinogenesis of GC and other digestive cancers.

## Data Availability Statement

Publicly available datasets were analyzed in this study. This data can be found here: TCGA-STAD, TCGA-COAD, TCGA-ESCA, TCGA-LIHC, TCGA-PAAD, and GSE84437.

## Author Contributions

Y-YL and WD designed the study. WD and JL implemented the data analysis. BL, QL, and QS provided the valuable suggestions. JL and WD drafted the manuscript. Y-YL revised the manuscript and coordinated the study. All authors read and approved the final manuscript.

## Conflict of Interest

The authors declare that the research was conducted in the absence of any commercial or financial relationships that could be construed as a potential conflict of interest.

## Footnotes

1 https://www.cancer.gov/about-nci/organization/ccg/research/structural-genomics/tcga

2 https://www.ncbi.nlm.nih.gov/geo/

3 https://cran.r-project.org/web/packages/survival/
