# Supplementary material for: Systematical Analysis of the Cancer Genome Atlas Database Reveals EMCN/MUC15 Combination as a Prognostic Signature for Gastric Cancer
Source: Front Mol Biosci. 2020 Feb 25;7:19. doi: 10.3389/fmolb.2020.00019 (PMC7055423; doi:10.3389/fmolb.2020.00019)
Supplement: Supplementary file 1 [file Table_1.DOCX]

Systematical Analysis of the Cancer Genome Atlas Database Reveals *EMCN*/*MUC15* Combination as a Prognostic Signature for Gastric Cancer

**Wentao Dai^1#^, Jixiang Liu^1#^, Bingya Liu^2^, Quanxue Li^1,3^, Qingqing Sang^3^, Yuan-Yuan Li^1*^**

^1^Shanghai Center for Bioinformation Technology & Shanghai Engineering Research Center of Pharmaceutical Translation, Shanghai Industrial Technology Institute, Shanghai, China

^2^Department of Surgery, Shanghai Key Laboratory of Gastric Neoplasms, Shanghai Institute of Digestive Surgery, Ruijin Hospital, Shanghai Jiao Tong University School of Medicine, Shanghai, China

^3^School of biotechnology, East China University of Science and Technology, Shanghai, China

*** Correspondence:**Dr. Yuan-Yuan Li
yyli@scbit.org

Supplementary Material


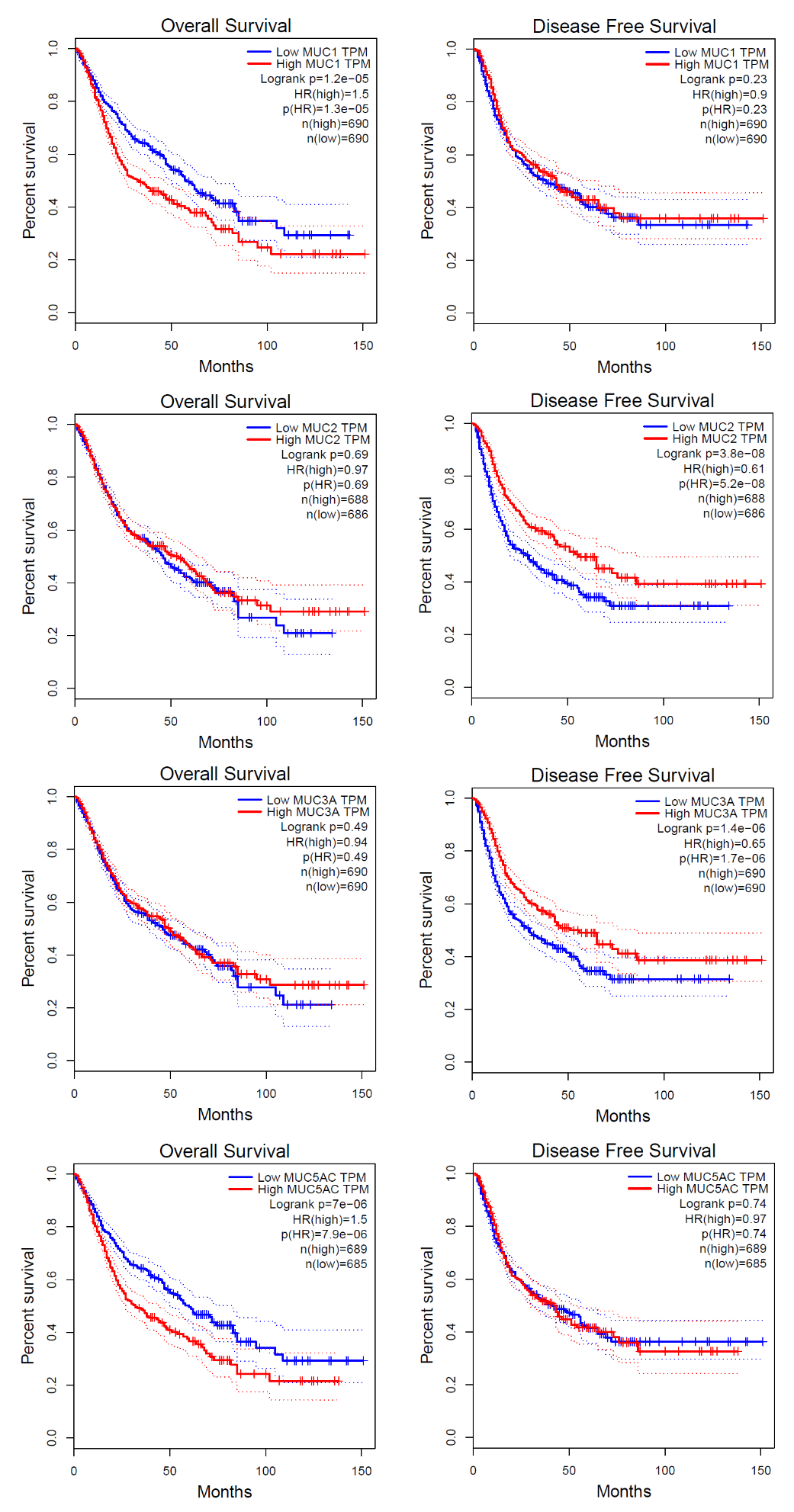


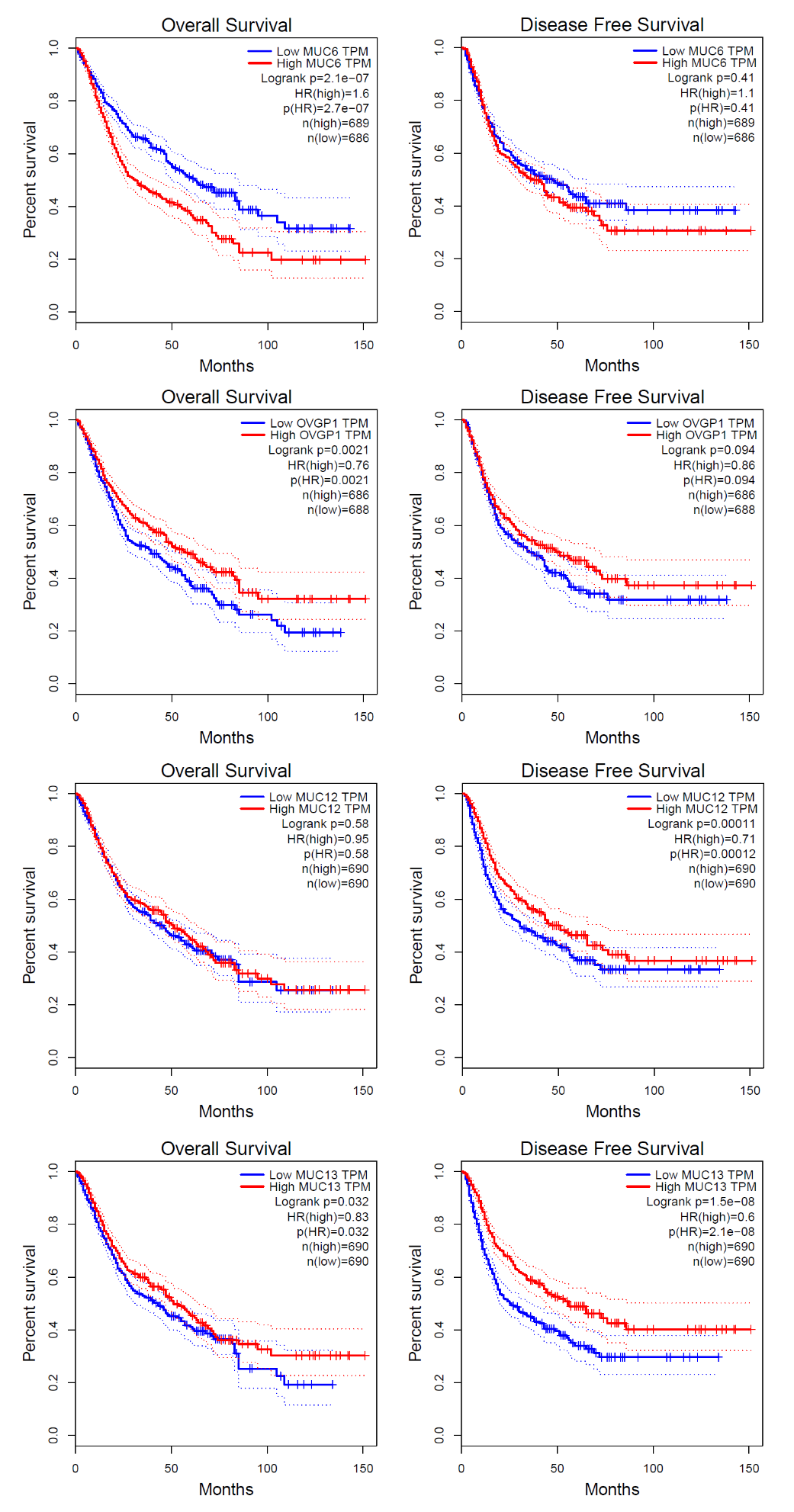


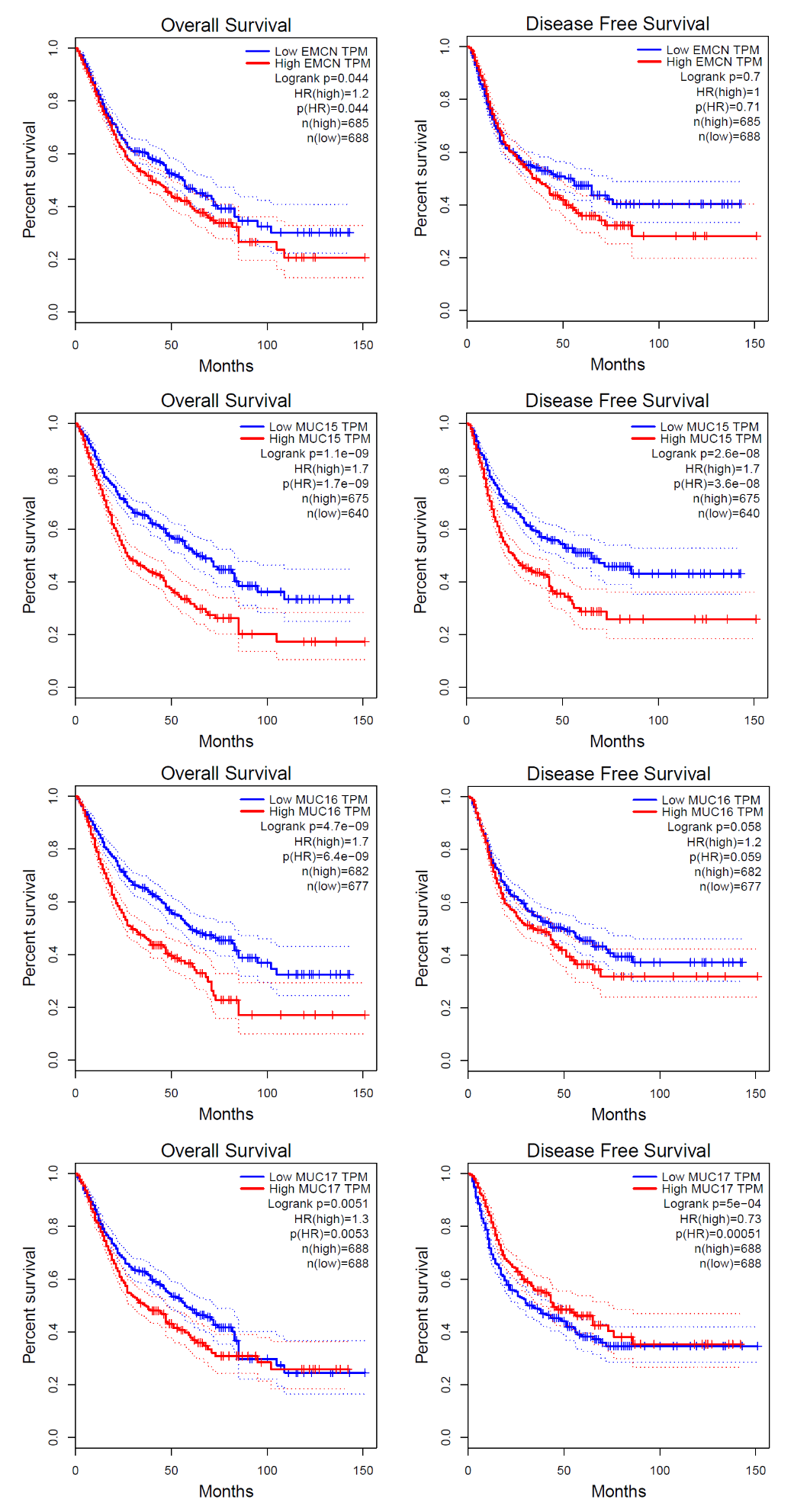


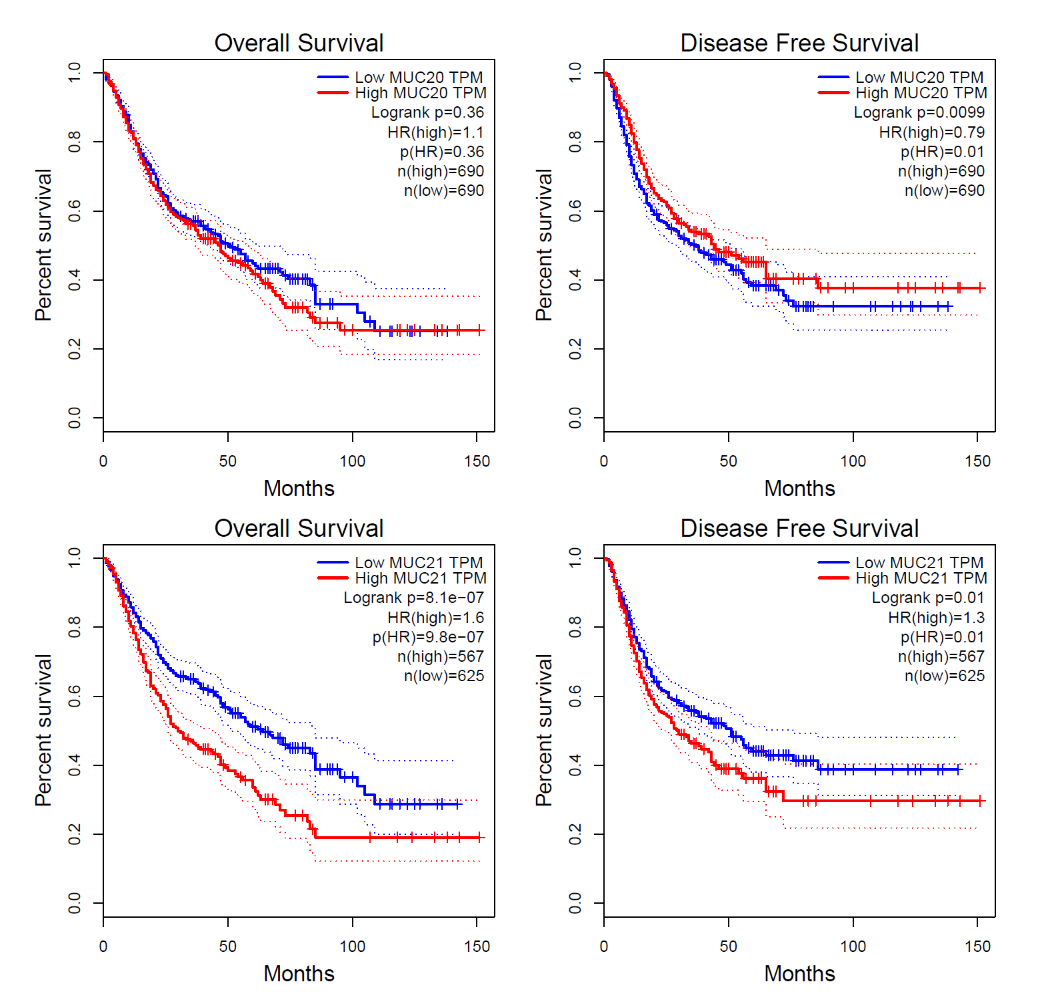


**Supplementary Figure 1.** Survival analysis of TCGA digestive cancer samples for MUC family genes. The 95% confidence intervals for survival time were shown as dotted lines in the Kaplan–Meier survival plot.

**Supplementary Table 1.** GO terms associated with EMCN and MUC15 top50 correlated genes

| **Pathway** | **p-value** | **Gene ID** |
| --- | --- | --- |
| Glomerulus vasculature development | 8.18E-06 | CD34/TEK/PECAM1/IFNG |
| Mitotic sister chromatid segregation | 9.57E-06 | CDCA5/CDCA8/ZWINT/KIF2C/CDT1/NCAPH/ESPL1 |
| Renal system vasculature development | 1.13E-05 | CD34/TEK/PECAM1/IFNG |
| Kidney vasculature development | 1.13E-05 | CD34/TEK/PECAM1/IFNG |
| Glomerulus development | 1.95E-05 | CD34/TEK/PECAM1/MEF2C/IFNG |
| Regulation of DNA binding | 2.67E-05 | CDCA5/HJURP/CDT1/IFNG/GZMA/  JAK2 |
| Mitotic metaphase plate congression | 6.76E-05 | CDCA5/CDCA8/KIF2C/CDT1 |
| Positive regulation of cell cycle phase transition | 7.85E-05 | CDCA5/CDT1/DTL/ESPL1/CDC25A |
| Inflammatory cell apoptotic process | 0.000148 | MEF2C/FAS/IFNG |
| Metaphase plate congression | 0.000196 | CDCA5/CDCA8/KIF2C/CDT1 |
| Sister chromatid segregation | 0.000204 | CDCA5/CDCA8/ZWINT/KIF2C/CDT1/NCAPH/ESPL1 |
| Response to interferon-gamma | 0.000229 | IRF1/NUB1/NLRC5/IFNG/CCL4/JAK2/ |
| Regulation of response to interferon-gamma | 0.000259 | NLRC5/IFNG/JAK2 |
| Regulation of interferon-gamma-mediated signaling pathway | 0.000259 | NLRC5/IFNG/JAK2 |
| Mitotic nuclear division | 0.000365 | CDCA5/CDCA8/ZWINT/KIF2C/CDT1/NCAPH/ESPL1 |
| Establishment or maintenance of cell polarity | 0.000387 | TEK/CDH5/KIF2C/MAP7/MARK1/  CCL4 |
| Chromosome, centromeric region | 0.000404 | CDCA5/CDCA8/ZWINT/KIF2C/  HJURP/CDT1 |
| Positive regulation of phosphatase activity | 0.000414 | MEF2C/IFNG/JAK2 |
| External side of plasma membrane | 0.000423 | CD34/S1PR1/CLEC14A/CDH5/FAS/  IFNG/FCGR3A |
| Chromosome segregation | 0.00046 | CDCA5/CDCA8/ZWINT/KIF2C/  HJURP/CDT1/NCAPH/ESPL1 |
| Side of membrane | 0.000709 | CD34/S1PR1/CLEC14A/GNG11/CDH5/FAS/IFNG/FCGR3A/JAK2 |
| Condensed chromosome | 0.000772 | CDCA5/ZWINT/KIF2C/HJURP/CDT1/NCAPH |


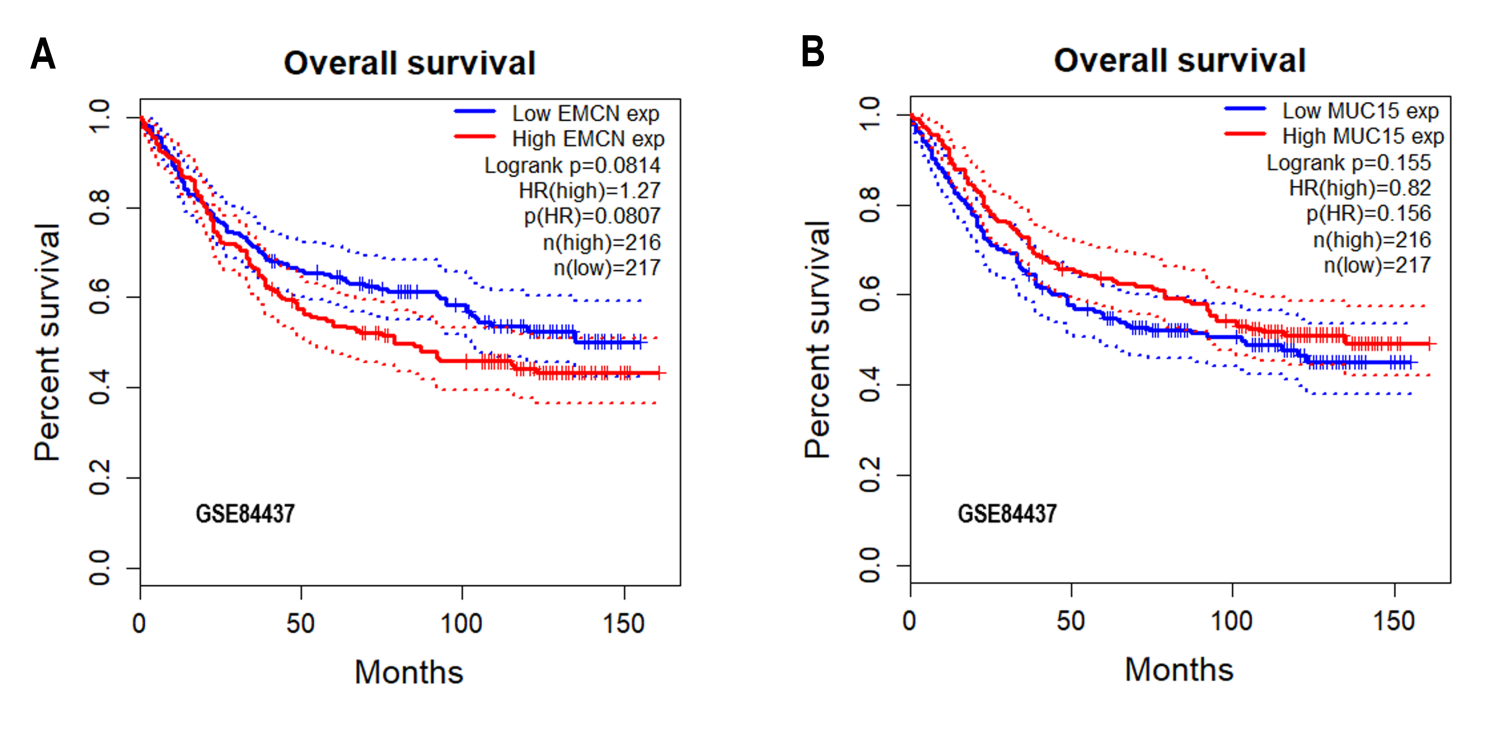


**Supplementary Figure 2.** Overall survival analysis of GSE84437 gastric cancer samples for *EMCN* (A) and *MUC15*(B). The 95% confidence intervals for survival time were shown as dotted lines in the Kaplan–Meier survival plot.
